# Tissue-preserving treatment with non-invasive physical plasma of cervical intraepithelial neoplasia—a prospective controlled clinical trial

**DOI:** 10.3389/fmed.2023.1242732

**Published:** 2023-08-15

**Authors:** Martin Weiss, Marcel Arnholdt, Anna Hißnauer, Irma Fischer, Birgitt Schönfisch, Jürgen Andress, Sophia Gerstner, Dominik Dannehl, Hans Bösmüller, Annette Staebler, Sara Y. Brucker, Melanie Henes

**Affiliations:** ^1^Department of Women’s Health, University of Tübingen, Tübingen, Germany; ^2^NMI Natural and Medical Sciences Institute, University of Tübingen, Reutlingen, Germany; ^3^Institute for Clinical Epidemiology and Applied Biometry, University of Tübingen, Tübingen, Germany; ^4^Department of Pathology and Neuropathology, University of Tübingen, Tübingen, Germany

**Keywords:** neoplasia, cervical intra epithelial neoplasia, physical plasma, non-invasive treatments, cervical cancer prevention

## Abstract

**Objective:**

Cervical cancer represents the fourth leading cause of cancer among women and is associated with over 311,000 annual deaths worldwide. Timely diagnosis is crucial given the lengthy pre-cancerous phase, which is typified by cervical intraepithelial neoplastic lesions. However, current treatment methods are often tissue-destructive and can be accompanied by severe side effects. To address these concerns, our study introduces a novel, gentle approach for the tissue-preserving treatment of CIN lesions.

**Results:**

We present findings of a controlled, prospective, single-armed phase IIb clinical trial performed at the Department for Women’s Health, Tübingen, Germany. From September 2017 to March 2022 we assessed 570 participants for study eligibility. Of the screened patients, 63 participants met with CIN1/2 lesions met the inclusion criteria and were treated with non-invasive physical plasma (NIPP). Assessment of treatment efficacy was based on a comprehensive analysis of histological and cytological findings, along with high-risk HPV infection load at 3 and 6 months post-treatment. Comparative analyses were performed retrospectively with data obtained from 287 untreated patients in the control group. Our findings indicate that patients treated with NIPP experienced an 86.2% rate of full remission, along with a 3.4% rate of partial remission of CIN lesions, which compares favorably to the control group’s rates of 40.4% and 4.5%, respectively. Additionally, we observed a twofold reduction in high-risk HPV infections following NIPP treatment. Minor side effects were observed, such as mild pain during treatment and short-term smear bleeding or increased vaginal discharge within 24 h after treatment. Given the experimental nature of NIPP treatment and the availability of established standard treatments, our study was designed as a non-randomized study.

**Conclusion:**

NIPP treatment offers a highly flexible and easy-to-apply method for treating pre-cancerous CIN1/2 lesions. This non-invasive approach is notable for its tissue-preserving nature, making it a promising alternative to current excisional and ablative treatments. CIN1/2 lesions were employed as preliminary *in vivo* models for the targeted treatment of CIN3 lesions.

**Clinical trial registration:**

https://www.clinicaltrials.gov, identifier NCT03218436.

## Introduction

Cervical intraepithelial neoplasia (CIN), the precursor of cervical cancer, is caused by persistent infection with human papillomavirus (HPV) (1). In 2018, 570,000 cases of cervical cancer and 311,000 deaths (90.0% of which occur in low- and middle-income countries) were registered worldwide, which makes it the fourth most common cancer in women (2, 3). CIN is categorized into classes of severity. While CIN1 is characterized by only mild dysplastic lesions, CIN2 and CIN3 present moderate and severe dysplastic lesions, respectively, including carcinoma *in situ*. Especially young women are at risk of developing cervical dysplasia. The age cohort of 25–29 years exhibits the highest recorded incidence rates of cervical intraepithelial neoplasia grade 2 (CIN2) and grade 3 (CIN3), with 8 cases per 1,000 women (4). The threat of developing cancer out of CIN and the invasive treatment procedures are associated with critical physical and emotional burdens on patients (3). In 2004, the World Health Organization (WHO) first published guidelines for clinicians regarding diagnosis and treatment of cervical dysplasia, with the last update in 2019 for the use of thermal ablation. For histologically confirmed CIN2/3 patients, the WHO currently suggests excisional treatments such as loop excision of the transformation zone (LLETZ) and under certain conditions (e.g., fully visible transformation zone and CIN lesions) destructive treatments like laser-, thermal-, and cryo-ablation (4). These currently established standard treatments represent invasive, tissue-destructive treatments, which usually require local or general anesthesia. Moreover, standard treatments may be associated with critical side effects, such as major bleeding, reduced fertility, and pregnancy complications (premature delivery, low birth weight, increased risk of cesarean section) (5, 6).

During the precancerous phase, which typically persists for several years before becoming invasive, effective management of early-stage disease may be achieved through the use of minimally invasive or non-invasive methods. Even undergoing excisional treatment, some cases show positive endocervical margins and HPV persistence being high-risk factors for CIN 3 recurrence within 5 years (7).

Low- and middle-income countries (LMICs) are disproportionately affected by cervical cancer, and limited medical supplies often hinder treatment. Conversely, in industrialized countries, the invasive nature of current standard procedures can lead to overtreatment. Therefore, developing new non-invasive therapeutic approaches is critical to address these issues.

Recently, a novel approach for treatment of precancerous cervical lesions is getting more attention. Non-invasive physical plasma (NIPP) is a tissue-preserving, highly adaptable solution for the treatment of precancerous and cancerous lesions, that can be easily adapted to the individual patient (8). Argon Plasma Coagulation (APC) can be used as a monopolar electrosurgical method in which the plasma beam develops under atmospheric pressure in an argon flow and follows the path of the least electrical resistance (9). Two electrodes are employed, with one serving as the active electrode and the other acting as a neutral “ground” electrode. The neutral electrode is positioned on the outer skin surface in close proximity to the targeted region of the body and is designed with a large contact area to prevent high current densities and subsequent skin burns. Between the active electrode and the neutral electrode, the current flows through the tissue, generating heat due to the Joule effect (10). While this effect may be disadvantageous for treating CIN lesions, it can be mitigated and restricted to ambient temperatures by adjusting the energy delivery and exposure duration. This is ensured by the homogeneous brush-like dynamic treatment as previously described (8, 11). On a molecular scale, NIPP gives rise to reactive oxygen and nitrogen species (ROS/RNS) (12, 13). In addition to its well-documented anti-cancer effects on various tumor types, NIPP has demonstrated promising transmucosal antineoplastic properties on cancer precursors, while maintaining the integrity of the underlying tissue structure (8, 14, 15). Within a monocentric dose-finding study we recently established the use of the VIO3/APC3 electrosurgical argon plasma device (Erbe Elektromedizin, Tübingen, Germany) for an effective but tissue-preserving *in vivo* application on CIN1/2 patients. Well-established HF electrosurgical argon plasma sources such as VIO3/APC3 have been widely available for clinical use for many years. The advantages of these devices include their high flexibility, sterile application probes, a wide range of potential clinical applications, and relatively low costs.

In the present prospective, monocentric, controlled clinical study, we validated the efficacy in 63 patients with histologically confirmed CIN 1/2 in comparison with the spontaneous remission rate of a control cohort of 287 patients. Based on the results of this study, prospective randomized controlled clinical trials may be conducted for CIN3 and further neoplastic diseases of mucosa in gynecology and beyond.

## Methods

### Study design

Here, we present findings of a controlled, prospective, single-armed phase IIb clinical trial (NCT03218436), performed at the Department for Women’s Health, Tübingen, Germany. The work described has been carried out in accordance with “The Code of Ethics of the World Medical Association” (Declaration of Helsinki) and was approved by the Ethical Committee of the Medical Faculty of the Eberhardt-Karls-University Tübingen (237-2017BO1). CIN was diagnosed by colposcopy-directed biopsy before investigational treatment. Patients were enrolled in the study after providing informed consent.

### Inclusion criteria

The key inclusion criteria for study participation were premenopausal women, 18–50 years of age, histologically confirmed CIN1/2, visibility of the entire transformation zone and the entire lesion margin. The key exclusion criteria were histologically confirmed CIN3 or invasive/micro-invasive disease, endocervical disease, severe inflammatory disease, other severe diseases, or pregnancy.

### Patient treatment

The patients underwent a clinical examination by colposcopy and visual inspection with acetic acid (VIA) and Lugol’s iodine staining to visualize CIN1/2 lesions, followed by NIPP treatment under colposcopic guidance using VIO3/APC3 and 3.2 mm APC probes (preciseAPC setting, effect 1) at a rate of 30 s/cm^2^, utilizing a reusable silicone electrode mat. The NIPP probe was passed over the tissue in defined “brush strokes” to avoid localized heating of the tissue. The treatment was carried out on an outpatient basis and without the need for either local or general anesthesia.

### Sample size

The primary endpoint for the confirmatory statistical analysis was the comparison of remission rates of CIN after 3 and 6 months between the NIPP and control group. The objective was to establish whether NIPP is superior to control within an absolute margin of 50.0% for the difference in remission rates. The calculation was performed for the one-sided confidence interval for relative risk (ratio of two proportions) with nQuery Advisor version 7.0, assuming a dropout rate of approximately 10.0%.

### Histology, cytology, and HPV assessment

Routine histochemical and immunohistological staining was performed on formalin-fixed, paraffin-embedded (FFPE) biopsy samples according to standard protocols at the Department for Pathology and Neuropathology at the Eberhard Karls University Tübingen. Routine staining of cytological smears was performed according to Papanicolaou (PAP) at the Immunocytology lab at the Department for Women’s Health at the Eberhard Karls University Tübingen. PAP smears were assessed according to the Munich III nomenclature. HPV testing was done using the Hybrid Capture 2 assay (HC2; Qiagen Inc., Hilden Germany) and p16 immunohistology (monoclonal antibody at 1:2,000 titration, Abcam Ab108349) at the Department of Medical Virology and the Department for Pathology and Neuropathology at the Eberhard Karls University Tübingen, respectively.

### Questionnaire and Freiburg index of patient satisfaction

Sensations of pain within 24 h following NIPP treatment were scored using a visual analog scale from 0 to 10. Scores of 0 and 1 were defined as “no pain,” 2–4 as “mild pain,” 5–7 as “moderate pain,” and 8–10 as “severe pain.” Other side effects were recorded as free text. Treatment satisfaction after NIPP intervention was assessed according to the “Freiburg index of patient satisfaction.”

### Study follow-up

Study participants were re-assessed for histopathological and cytological remission 3 and 6 months following NIPP treatment. For this, a clinical examination by colposcopy and VIA (4.0% acetic acid and Lugol’s iodine staining) as well as colposcopy-directed biopsy was performed by trained and certified gynecologists.

### Statistical analysis

Statistical comparisons between independent samples (control group vs. NIPP group), as well as comparisons within a group (control vs. control or NIPP vs. NIPP), were performed using the asymptotic Wilcoxon test. To compare HPV infection status before and after treatment, the McNemar test was employed. All tests were carried out using SPSS 16.0 (SPSS Inc., Chicago, United States), with *p*-values of <0.05 considered statistically significant.

## Results

**Figure 1:**
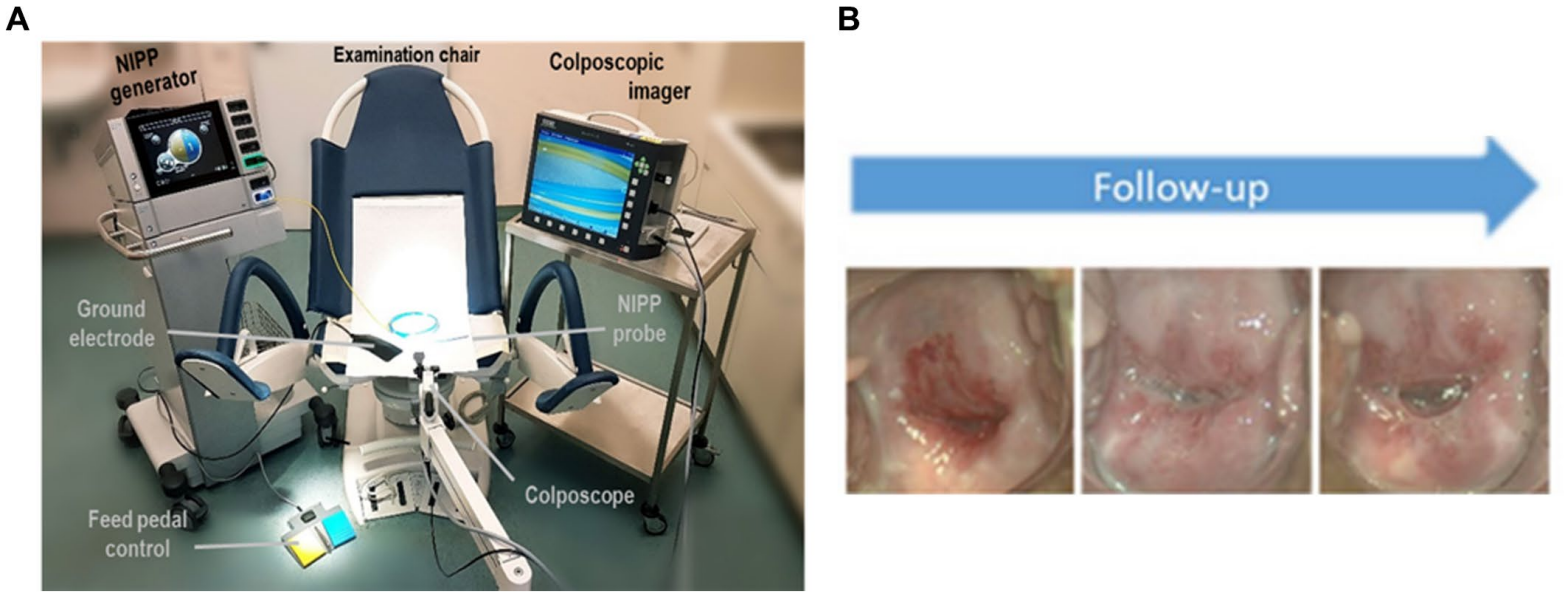
**(A)** Setup for NIPP patient treatment **(B)** Representative images of the portio after 2, 12, and 24 weeks of treatment. Figures taken from Marzi et al. (8).

**Figure 2:**
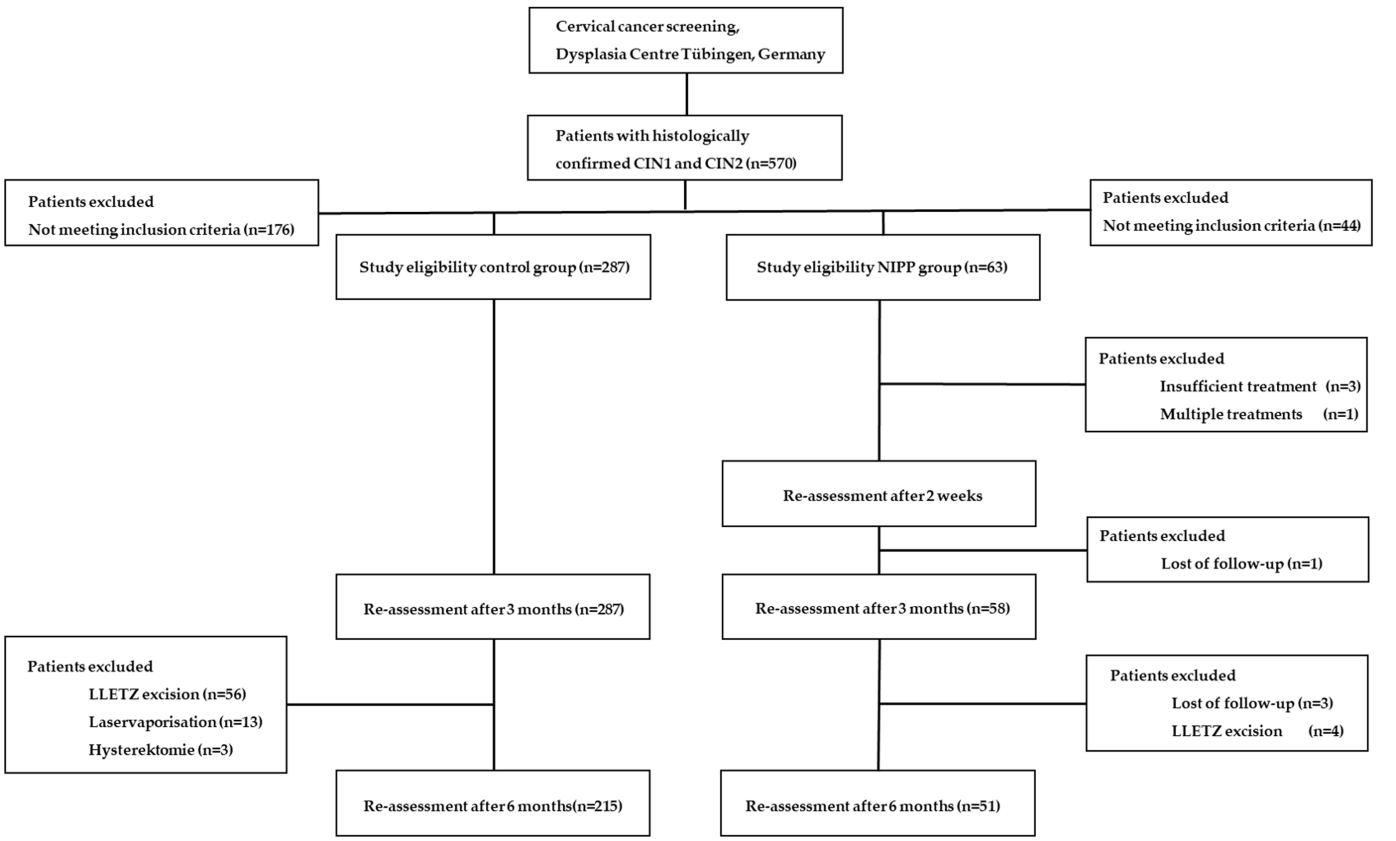
Trial flow chart of patient recruitment and study visits.

From September 2017 to March 2022, we assessed 570 participants for study eligibility. NIPP was prospectively applied in 63 patients with histologically proven CIN 1/2 in a controlled clinical trial (NCT03218436) at the Dysplasia Center of the Department for Women’s Health, Tübingen, Germany (Figures 1A, B). 59 (94.0%) participants completed 6 months of treatment and were eligible for 6-month follow-up, of whom 51 (81.0%) attended the 6-month follow-up examination (Figure 2). The outcome of NIPP treatment was compared to the spontaneous remission rate of 287 participants in the control group. In both groups, CIN was diagnosed by colposcopy-directed biopsy prior to study enrollment.

### Patient characteristics

The key inclusion criteria for study participation were premenopausal women, 18–55 years of age, with histologically confirmed CIN1/2, visibility of the entire transformation zone (T1/2) and the entire lesion margin. Key exclusion criteria included CIN3 or invasive disease, endocervical involvement, signs of severe inflammation, pregnancy, and any severe secondary diseases. Indication for NIPP treatment was only given in case of either persisting lesions over 24 months or by the patient’s compelling wish for therapy (urgent desire to conceive, severe anxiety, and/or significant psychological stress). Since CIN1/2 lesions are precursors of the target lesion CIN3, they were utilized as a preliminary *in vivo* model to assess the potential efficacy of NIPP treatment for CIN3. 58.7% of patients showed CIN1/2 lesions older than 24 months and therefore had an indication for ablative treatment. Around 20.0% of patients showed CIN1/2 lesions persisting between 6 and 12 months and 12 and 24 months, respectively. In these specific patients, ablative treatment was recommended due to their urgent desire to conceive or significant psychological distress, which prompted their strong desire for therapy. Patients were informed about the experimental nature of NIPP treatment, for which there was no clinical evidence of the therapeutic benefit at time of treatment. Furthermore, patients were informed about other existing and clinically well-established standard treatment options (such as laser and LLETZ). Before NIPP treatment, the patients provided written informed consent in accordance with the approved ethical protocol (237-2017BO1) and were informed about the experimental nature of the treatment, as well as the availability of other clinically established standard treatments such as laser ablation and LLETZ. The individual CIN lesions were visualized using colposcopy and VIA and then treated with NIPP under colposcopic guidance, without the use of local or general anesthesia (movie S1). Histopathological and cytological remission was assessed after 12 and 24 weeks.

The significant difference in the comparison groups was due to the prospective non-randomized nature of the study.

Table 1 shows patient characteristics of the control group (206 patients (72.0%) with CIN 1 and 81 patients (28%) with CIN 2) and the NIPP group (23 patients (36.0%) with CIN 1 and 40 patients (64.0%) for CIN 2). The significant difference in the comparison groups was due to the prospective non-randomized nature of the study.

**Table 1:** Patient characteristics before study enrollment.

		NIPP			Control		
		CIN1	CIN2	Total	CIN1	CIN2	Total
Histological characterization (*n*, %)		23 (36.0%)	40 (64.0%)	**63 (100.0%)**	206 (72.0%)	81 (28.0%)	**287 (100.0%)**
Age, years (*n* = 63, mean, range)		31.7	29.1	**30.1 (21–59)**	36.3	30.5	**34.7 (18–76)**
Gravidities (*n* = 51, mean)		0.7 (*n* = 17)	0.7 (*n* = 34)	**0.7**	−	−	**−**
Parities (*n* = 51, mean)		0.7 (*n* = 17)	0.6 (*n* = 34)	**0.7**	−	−	**−**
Time diagnosis to treatment (*n*, %)	>3 months	22 (95.7%)	39 (100.0%)	**61 (98.3%)**	−	−	**−**
	>1 year	16 (84.2%)	33 (86.8%)	**49 (77.7%)**	−	−	**−**
	>2 years	12 (63.2%)	25 (65.8%)	**37 (58.7%)**	−	−	**−**
Cytology (*n*,%)	PAP II-a	7 (30.4%)	9 (22.5%)	**16 (25.4%)**	38 (18.6%)	11 (13.6%)	**49 (17.2%)**
	PAP II-p	3 (13.0%)	2 (5.0%)	**5 (7.9%)**	35 (17.2%)	6 (7.4%)	**41 (14.4%)**
	PAP IIID1	10 (43.5%)	6 (15.0%)	**16 (25.4%)**	56 (27.5%)	26 (32.1%)	**82 (28.8%)**
	PAP IIID2	3 (4.8%)	20 (50.0%)	**23 (36.5%)**	55 (27.0%)	32 (39.5%)	**87 (30.5%)**
	PAP III-p	0 (0.0%)	3 (7.5%)	**3 (4.8%)**	20 (9.8%)	3 (3.7%)	**23 (8.1%)**
HPV high risk (*n*, %)	Negative	9 (39.1%)	7 (17.5%)	**16 (25.4%)**	−	−	**−**
	Positive	14 (60.9%)	33 (82.5%)	**47 (74.6%)**	−	−	**−**

Bold values in table are meant to emphasize the combined (total) values of CIN1 and CIN2 sub-groups.

At study entry, 25.4% of the NIPP group and 17.2% of the control group had normal cytological results and were categorized as PAP II-a. Of the NIPP group, 66.7% of participants were classified as group III, compared to 67.4% of the control cohort. In the NIPP group, 74.6% of patients displayed infections with high-risk HPV strains. Data regarding gravidity and parity as well as HPV typing were collected exclusively in the NIPP group.

Figure 1 presents the setup used for NIPP treatment as well as representative images of the portio over the course of the study period.

### Histological assessment of NIPP efficacy

Table 2 shows NIPP-related and spontaneous remission proportions of CIN1/2 lesions. It is important to note that despite being distinct pathological entities, Table 2 consolidates both low-grade and high-grade CIN lesions for better clarity. For detailed information on sub-group data regarding CIN1 and CIN2, please refer to Supplementary Table S1. Treatment success was evaluated after 3 and 6 months, respectively. Histological characterization was assessed by colposcopy-directed tissue biopsy.

**Table 2:** Histological remission rates after NIPP treatment.

		After 3 months		After 6 months	
		NIPP	Control	NIPP	Control
Histological characterization (*n*,%)	No CIN	50 (86.2%)	116 (40.4%)	44 (80.0%)	129 (44.9%)
	CIN1	2 (3.4%)	87 (30.3%)	2 (3.6%)	46 (16.0%)
	CIN2	4 (6.9%)	55 (19.2%)	2 (3.6%)	27 (9.4%)
	CIN3	2 (3.4%)	29 (10.1%)	3 (5.5%)	13 (4.5%)
CIN changes to study start	Full remission	50 (86.2%)	116 (40.4%)	44 (80.0%)	129 (44.9%)
	Partial remission	2 (3.4%)	13 (4.5%)	1 (1.8%)	7 (2.4%)
	Persistence	4 (6.9%)	100 (34.8%)	3 (5.5%)	50 (17.4%)
	Progression	2 (3.4%)	58 (20.2%)	3 (5.5%)	29 (10.1%)

Both the NIPP and control group showed significant remission rates 3 and 6 months after study entry (*p* < 0,001, determined by Wilcoxon test). In the NIPP group, 50 of 58 patients did not display any abnormal histological results after 3 months, which corresponds to a complete remission rate of 86.2%, which was significantly higher than observed in the control group (40.4%, *p* < 0.001, determined by Fishers exact test). CIN progression was observed in 2 NIPP treated participants (3.4%), but in 58 participants (20.2%) of the control group. At 6 months follow up the rate of NIPP patients with normal histological results slightly decreased to 44 (80% remission), compared to the control group (129 participants, 44.9% remission). To reflect NIPP effectiveness at 6 months, the same denominator was used as at 3 months. Again, with a 10.1% progression rate and 17.4% persistence rate the control group was inferior to that of NIPP-treated patients (5.5% each). All changes in remission rates between 3 and 6 months were not statistically significant. Intervention by LLETZ excision, laservaporisation, or hysterectomy took place at the specific request of the patients 6 months after study entry. This was the case for 4 (7.8%) study participants in the NIPP group, whereas 72 (25.1%) participants in the control group opted for intervention. Furthermore, remission rates between the specific sub-groups (CIN1/CIN2) were significantly higher in the NIPP group (3 months complete remission rate, CIN1, NIPP: 95.2%, control: 46.1%; CIN2, NIPP: 81.1%, control: 25.9%; 6 months complete remission rate, CIN1, NIPP 85.7%, control: 51.9%; CIN2, NIPP: 76.5%, control: 27.2%; Supplementary Table S1).

### Cytological assessment of NIPP efficacy

For cytological assessment, PAP smear tests were performed 3 and 6 months after study enrollment. Results of the study visits are summarized in Table 3.

**Table 3:** Cytological results at study entry, 3 and 6 months follow-up.

		Study entry		After 3 months		After 6 months	
		NIPP	Control	NIPP	Control	NIPP	Control
Cytology (*n*, %)	PAP II-a	16 (25.4%)	49 (17.2%)	31 (53.4%)	80 (28.4%)	30 (54.5%)	101 (35.8%)
	PAP II-p	5 (7.9%)	41 (14.4%)	12 (20.7%)	40 (14.2%)	12 (21.8%)	36 (12.8%)
	PAP IIID1	16 (25.4%)	82 (28.8%)	4 (6.9%)	73 (25.9%)	3 (8.8%)	40 (14.2%)
	PAP IIID2	23 (36.5%)	87 (30.5%)	9 (15.5%)	61 (21.6%)	2 (3.6%)	19 (6.7%)
	PAP III-p	3 (4.8%)	23 (8.1%)	1 (1.7%)	11 (3.9%)	2 (3.6%)	7 (2.5%)
	PAP IV-a	−	−	1 (1.7%)	17 (6.0%)	2 (3.6%)	8 (2.8%)

At 3 months follow-up, the average PAP smear results significantly improved in both cohorts (*p* < 0.001, determined by Wilcoxon test). In line with this, NIPP treated patients PAP smear tests were inconspicuous in 53.4% of cases, compared to 28.4% of the control group. For a small number of patients (NIPP: 1.7%, control: 6.0%) a worsening of cytological findings to PAP IV-a was observed. After 6 months, both groups showed an increased number of patients with normal PAP II-a-categorized smear results compared to study entry (NIPP: 54.5%, control: 35.8%). The number of participants with group III PAP results decreased for both NIPP and control (NIPP: 16.0%, control: 23.4%). Overall, within the NIPP group, 29 patients (52.7%) showed improved cytological findings compared to study entry. Twelve patients (21.8%) showed persistence of findings after 6 months and 14 patients (25.5%) showed an aggravation of cytological findings. In 107 patients (37.9%) of the control group, the findings improved after 6 months compared to baseline. 73 patients (25.8%) had unchanged cytological findings and 102 patients (36.1%) had a progression of findings. Remission rates for the specific sub-groups (CIN1/CIN2) are displayed in Supplementary Table S2.

### High-risk HPV infection rates following NIPP treatment and assessment of patient comfort

There are more than 200 known human papillomaviruses, only some of which are classified as oncogenic high-risk variants (16). In this study we investigated the profile of HPV types in patients of the NIPP group at study entry and 6 months after NIPP treatment (Table 4).

**Table 4:** HPV infection type characterization before and 6 months after NIPP treatment.

		Study entry	6 months
HPV high risk (*n*, %)	Negative	16 (25.4%)	30 (62.5%)
	Positive	47 (74.6%)	18 (37.5%)
HPV low risk (*n*,%)	Negative	46 (86.6%)	31 (86.1%)
	Positive	7 (13.2%)	5 (13.9%)

Prior to treatment, 74.6% of patients were infected with high risk HPV strains. Examination after 6 months showed an overall decrease of high-risk HPV infections by 2-fold which proved to be statistically significant (*p* < 0.001, McNemar test). Considering the CIN1 and CIN2 subgroups, the decrease within the CIN1 group was not significant (2-fold decrease, *p* = 0.063) in contrast to the CIN2 (1.9-fold decrease, *p* = 0.003) group, taking into account that the group size of CIN2 is almost double. The data of the sub-groups can be taken from Supplementary Table S3. During the course of the study, the participants were asked about their perception of pain and their satisfaction with the treatment according to the Freiburg Index of Patient Satisfaction. Results of the survey are summarized in Table 5. Across the patients who received NIPP treatment, no acute dose-limiting toxicities were observed. A total of 55.0% of the patients (*n* = 33) experienced mild adverse events (grade 1), imaged by short-term smear bleeding and increased vaginal discharge, within the first 24 h after NIPP treatment. The most common side effect was mild to moderate local discomfort, including pain and cramping, during NIPP treatment. All adverse events mentioned resolved spontaneously without treatment.

**Table 5:** Survey of patient satisfaction according to the Freiburg Index of Patient Satisfaction and pain perception.

Intensity of pain or discomfort (*n* = 60)	No pain	Mild pain	Moderate pain	Severe pain		
Prior treatment	54 (90%)	4 (7.6%)	2 (3.3%)	−		
During treatment	21 (35.0%)	26 (43.3%)	10 (16.7%)	3 (5.0%)		
After 4 h	32 (53.3%)	19 (31.7%)	8 (13.3%)	1 (1.7%)		
After 2 days	51 (85.0%)	7 (11.7%)	1 (1.7%)	1 (1.7%)		
After 1 week	50 (84.7%)	5 (8.5%)	2 (3.4%)	2 (3.4%)		
**Any side effects perceived (*n* = 60)**						
Yes	33 (55.0%)					
No	27 (45.0%)					
**Treatment was not perceived as stressful (*n* = 59)**	**Agree very strongly**	**Agree strongly**	**Agree**	**Disagree**	**Disagree strongly**	**Disagree very strongly**
	27 (45.8%)	21 (35.6%)	8 (13.6%)	2 (3.4%)	1 (1.7%)	−
**Recovery from treatment was rapid (*n* = 59)**	**Agree very strongly**	**Agree strongly**	**Agree**	**Disagree**	**Disagree strongly**	**Disagree very strongly**
	38 (64.4%)	14 (23.7%)	6 (10.2%)	−	−	1 (1.7%)
**Treatment was perceived as success (*n* = 54)**	**Agree very strongly**	**Agree strongly**	**Agree**	**Disagree**	**Disagree strongly**	**Disagree very strongly**
	16 (29.6%)	31 (57.4%)	6 (11.1%)	1 (1.9%)	−	−
**Would repeat the treatment (*n* = 60)**	**Agree very strongly**	**Agree strongly**	**Agree**	**Disagree**	**Disagree strongly**	**Disagree very strongly**
	40 (66.7%)	16 (26.7%)	2 (3.3%)	2 (3.3%)	−	−
**Overall rating of the treatment (*n* = 58)**	**Excellent**	**Very good**	**Good**	**Satisfactory**	**Bad**	**Very bad**
	18 (31.0%)	33 (55.2%)	7 (12.1%)	1 (1.7%)	−	−

## Discussion

In this monocentric, prospective study we aimed to evaluate the effectiveness of a single NIPP treatment for CIN1 and CIN2 lesions in adult women, compared to the spontaneous remission of these lesions within a 24-week follow-up period. Our results show that NIPP treatment significantly improved both histological and cytological remission of CIN, and resulted in a significant decrease in high-risk HPV status. Moreover, NIPP treatment was easily performed without the need for anesthesia and enabled tissue preservation, making it a promising approach for cancer prevention.

Cervical cancer is a public health problem, as recognized by the WHO. The executive board of the WHO called for action in January 2019 and requested the director general as well as member states and stakeholders to develop a global strategy against CC, which includes vaccination, screening, and treatment (17). The currently defined strategy pursues the 90-70-90 goal, which means that by 2030 90.0% of girls should be fully vaccinated against HPV, 70.0% of women are screened by the age of 35, at the age of 45, and 90.0% of women identified with cervical disease receive treatment.

Technological advances provide a possibility to contribute to the acceleration of cervical cancer elimination. Less invasive treatment strategies are not only under clinical evaluation for early stage cervical cancer diseases but also for precancerous lesions of the cervix uteri (18, 19). In the hereby presented study, we give evidence for effective treatment and disease control of precancerous CIN lesions with NIPP treatment using a next-generation electrosurgical argon plasma device. In contrast to other conventional treatments like LLETZ, as well as cryo- and thermo ablation the novel and innovative NIPP concept enables anesthesia-independent, tissue-preserving, and easily performable cancer prevention. The conventional treatment for high-grade CIN typically involves local excision of the lesion, which requires local or general anesthesia. The surgical management of CIN has been linked to a heightened risk of preterm delivery, low birth weight, and preterm premature rupture of membranes before 37 weeks of pregnancy, particularly in cases involving cold knife conization and LLETZ procedures in several studies and systematic reviews (5, 6, 20). Additionally, the risk of PD was found to be influenced by factors such as the size of the cone biopsy, cervical length, repeated treatment, and a short interval between conization and subsequent pregnancy. Also, the number of possible repetitions is limited due to the considerable loss of tissue mass. Future investigations should aim to assess the obstetric outcomes following NIPP treatment once a substantial cohort of patients has undergone the novel procedure and subsequently delivered.

Thermal approaches with low temperature (cryoablation, −70°C or lower) or high temperature (heat ablation, at least 100°C) induce necrotic tissue destruction and are WHO-recommended procedures in low- and middle-income countries (LMICs). But the need for a refrigerant gas (cryotherapy), the time-consuming nature (multiple overlapping applications), and restricted dimensions of pre-defined thermoprobes result in costly difficulties for the introduction of both procedures in LMIC infrastructures (21). Thermal ablation procedures, such as NIPP treatment, do not allow for obtaining definitive histology, which necessitates control examinations. This is a disadvantage when compared to excisional procedures.

The NIPP procedure was carried out using a modular high-frequency device, which is highly available in surgical facilities. A down-sized single-mode and battery-driven generator powered by solar panels could extend the use of this new therapeutic approach to LMICs and is currently under development. NIPP has been demonstrated to induce tissue-preserving molecular and cellular responses in cervical cancer cells and all mucosal tissue layers of CIN, in contrast to conventional physical procedures for tissue destruction. Several studies have shown that NIPP affects cellular processes such as cell growth, metabolism, cell cycle, DNA integrity (8, 22), and apoptosis primarily through the generation of reactive oxygen species (ROS) (23–24). In this study NIPP treatment showed to be highly flexible and individually adaptable to the size and shape of the lesion. One single *in vivo* application of NIPP was sufficient to achieve full histological remission in 86.2% and improvement of cytological findings in 52.7% of patients. Both, histological and cytological assessment was blinded. The observed difference between the histologic and cytologic findings could potentially be attributed to the development of radiation-induced atypia, which is a known phenomenon associated with irradiation and may contribute to misinterpretation of PAP smears (25). Infection with high risk HPV strains was significantly reduced 6 months after treatment. Pinder et al. are currently conducting a trial (NCT02956239) to compare the efficacy of heat ablation, cryoablation, and LLETZ (21). In the pilot phase, treatment success rates were reported as 60.0% for cryoablation, 64.0% for heat ablation, and 67.0% for LLETZ, with success defined as either HPV type-specific clearance and/or negative visual inspection. However, these measures may be insufficient and overstate the efficacy, as CIN diseases can affect and persist in deeper cervical glands.

The present study has a major strength in its use of colposcopy-directed biopsy to determine histological remission rates, which is a more reliable method than those used in previous studies of CIN treatments. However, the study is limited by its non-randomized design, which was intended to allow for maximum patient autonomy and voluntariness. Additionally, patients who received interventional treatments were excluded from both groups, which may have introduced bias. The exclusion of a higher number of patients in the control group due to LLETZ or laservaporisation may have resulted in a relative overperformance of the control group. Additionally, it should be noted that this study only focused on CIN1 and 2 lesions, whereas CIN3 is the actual target lesion. The reason for this was to investigate the fundamental efficacy of NIPP on CIN compared to spontaneous remission without treatment. CIN2, similar to CIN3, is classified as a high-grade lesion; however, it can be managed in accordance with established guidelines through the implementation of destructive therapeutic interventions, whereas CIN3 necessitates excisional treatment via LEETZ as the optimal approach. Hence, CIN2 represents a specifically suitable stage of dysplasia, characterized by high-grade abnormalities, for the investigation of NIPP effects. In the future, further studies are needed to compare the effectiveness of NIPP on CIN3 with standard treatments. An inclusion criterion for participation in the study was a completely visible transformation zone (T1 or T2). As is the case for other ablative or excisional treatment procedures, we observed a trend of shifting of the transformation zone to T3, which was the case in 70.6% of the patients. This shift could impede colposcopic examination thereafter. Only 1 patient (2.0%) showed a shift of the transformation zone from T2 to T1, while no change of the transformation zone occurred in 27.5% of participants.

Due to its highly flexible and patient-specific application, NIPP enabled side effect-free and nearly painless treatment of CIN1/2 without major postintervention complications. In contrast, LLETZ has a reported 9.7% rate of complications, including abdominal pain, vaginal bleeding, vaginal discharge, bladder spasms, and in some cases major complications like bowel injury and hemorrhage. In addition, surgical intervention leads to cervical stenosis in 2.1% of patients, making follow-up cytology extremely painful to impossible (26, 27).

Although NIPP mainly affects cancerous and pre-cancerous cells, adverse side effects like DNA and lipid peroxidation can occur in healthy cells due to excess of RONS (23). DNA damage has been frequently shown to be associated with NIPP treatment. Despite the significant induction of RONS with subsequent chemical modifications of DNA molecules, no genotoxic NIPP effects have been demonstrated so far (28). Finding an appropriate plasma dose is crucial to achieve the desired therapeutic effect without harming surrounding tissue. In the future simultaneous dose-tracking may be established to further reduce tissue harming and side effects.

## Data availability statement

The original contributions presented in the study are included in the article/Supplementary material, further inquiries can be directed to the corresponding author.

## Ethics statement

The studies involving humans were approved by Ethical Committee of the Medical Faculty of the Eberhardt-Karls-University Tübingen (237-2017BO1). The studies were conducted in accordance with the local legislation and institutional requirements. The participants provided their written informed consent to participate in this study. Written informed consent was obtained from the individual(s) for the publication of any potentially identifiable images or data included in this article.

## Author contributions

MW and MA discussed the results and wrote the paper. MW, HB, AS, and MH conceived the strategy and designed the experiments. MW and MH performed the experiments and designed and performed the clinical study. MW, MA, AH, SG, IF, and BS analyzed the data. DD, SG, JA, and SB reviewed the manuscript. All authors contributed to the article and approved the submitted version.

## Funding

This research was funded by German Research Foundation (project no. 501640545 to MW; Graduate School 2543/1 “Intraoperative Multi-Sensory Tissue-Differentiation in Oncology” (project(s) A3 and C2) to GRK 2543/1 to SB and MW; 04/2020). This work was supported by Erbe Elektromedizin GmbH, Tübingen (loaner of the NIPP device and equipment but did not play any role in the study design, collection, analysis, interpretation of the data, writing of the article or the decision to submit it for publication).

## Conflict of interest

The authors declare that the research was conducted in the absence of any commercial or financial relationships that could be construed as a potential conflict of interest.

## Publisher’s note

All claims expressed in this article are solely those of the authors and do not necessarily represent those of their affiliated organizations, or those of the publisher, the editors and the reviewers. Any product that may be evaluated in this article, or claim that may be made by its manufacturer, is not guaranteed or endorsed by the publisher.
