# Supplementary material for: Tissue-preserving treatment with non-invasive physical plasma of cervical intraepithelial neoplasia—a prospective controlled clinical trial
Source: Front Med (Lausanne). 2023 Aug 15;10:1242732. doi: 10.3389/fmed.2023.1242732 (PMC10465690; doi:10.3389/fmed.2023.1242732)
Supplement: Supplementary file 1 [file Table_1.DOCX]

**S1 table: Histological remission rates of CIN1 and CIN2 sub-groups 3 and 6 months after study enrollment.**

**S2 table: Cytological results of PAP smear tests of CIN1 and CIN2 sub-groups 3 and 6 months after study entry**

**S3 table: HPV infection status of CIN1 and CIN2 sub-groups before and 6 months after NIPP treatment**
